# An interpretable machine learning framework for adverse drug reaction prediction from drug-target interactions

**DOI:** 10.1371/journal.pone.0340900

**Published:** 2026-01-30

**Authors:** Joseph Roberts-Nuttall, Alan M. Jones, Marco Castellani, Duc Pham

**Affiliations:** 1 School of Mechanical Engineering, University of Birmingham, Edgbaston, United Kingdom; 2 School of Pharmacy, University of Birmingham, Edgbaston, United Kingdom; National University, SUDAN

## Abstract

**Background:**

Adverse drug reactions (ADRs) present challenges to patient safety and healthcare systems. Current pharmacovigilance methods, such as the Yellow Card Scheme (YCS), provide valuable post-marketing data, but the mechanistic causes of these ADRs are not fully understood. Leveraging drug-target interaction data with interpretable machine learning offers a promising approach to anticipate ADRs and understand their underlying mechanisms.

**Objective:**

This study proposes an interpretable machine learning (ML) framework to predict significant ADRs using drug-target interaction data. The framework aims to identify key pharmacological relationships, helping to inform drug safety.

**Methods:**

Drug-target interaction data from STITCH was combined with ADR reports from the YCS. Disproportionality analysis identified significant ADR signals which were used to train Random Forest classifiers across System Organ Class (SOC) categories. Class imbalance was addressed with SMOTE and Tomek, and Bayesian optimisation refined hyperparameters. Feature importance scores provided interpretability, and the top features were validated using known target-disease associations from DisGeNET.

**Results:**

Prediction performance varied across SOC categories, with ROC AUC scores up to 0.94. Feature importance analysis identified pharmacologically relevant targets, validated using DisGeNET and comparisons with SIDER highlighted the added value of real-world data.

**Conclusions:**

The interpretable ML framework links drug-target interactions to ADRs, offering a promising approach for predictive pharmacovigilance (PPV) and supporting safer drug development.

## 1 Introduction

Adverse drug reactions (ADRs) are unwanted or harmful reactions that occur when a drug is administered correctly, at the recommended dose, to the appropriate patient, and for its intended purpose [1]. ADRs pose a significant challenge to healthcare, affecting patient health, quality of life (QoL), and generating a substantial health economic strain. In the UK, ADRs account for ~16.5% of all in-patient hospital admissions and cost the NHS ~ £2.2 billion annually [1]. ADRs are commonly classified using the Rawlins-Thompson classification system into Type A and Type B. Type A reactions are dose dependent, predictable and typically arise from on-target pharmacological effects, occasionally involving related off-target actions [2]. These account for around 40% of ADR related admissions and are largely preventable [1]. Type B reactions, accounting for ~60% remaining, are not dose-dependent and are unpredictable. They are often immune-mediated or idiosyncratic and may arise from off-target mechanisms not captured by conventional pharmacological understanding of how the drug works [3]. As such, they have traditionally been considered unavoidable. However, by incorporating drug-target interaction data, including both on-target and off-target effects, there is increasing potential to identify mechanistic signals underlying both types of ADRs, thereby expanding the scope of what can be predicted and potentially prevented.

Pharmacology examines how drugs interact with biological systems, encompassing both therapeutic effects and harmful outcomes, such as toxicity and ADRs. It includes pharmacodynamics, which explores the biological effects of drugs and their mechanisms of action, and pharmacokinetics, which studies how the body absorbs, distributes, metabolises and excretes drugs [4]. While drugs are designed to interact with specific targets, they can also interact with unintended targets. These unintended interactions can trigger adverse effects, contributing to the avoidable ADRs seen in clinical settings [5]. Understanding unintended interactions may help reveal their underlying causes and support more effective prevention of ADRs. Pharmacology is therefore central to identifying, predicting and managing drug-related harm. In this report, the term target refers to both proteins and the genes that encode them. Drugs primarily interact with proteins, but identifying targets at the gene encoding level enables deeper analysis of drug response variability, and to avoid confusion, both have been standardised under the term ‘target.’

Monitoring of ADRs is achieved through retrospective pharmacovigilance. The World Health Organisation defines pharmacovigilance as the science and practice of detecting, assessing, understanding and preventing adverse effects or medicine-related problems [6]. Current pharmacovigilance relies on post-marketing surveillance and statistical analysis of reported, suspected drug-event combination (DEC) side-effects. The detection of significant ADRs signals can lead to restricted drug use, updated patient information leaflets (PIL), National Institute for Health and Care Excellence (NICE) guidelines, summaries of product characteristics (SmPC) or infrequently, market withdrawal [7]. The thalidomide disaster, where a morning sickness drug caused severe birth defects, leading to market withdrawal in 1961, prompted the creation of the Yellow Card Scheme (YCS) in 1964 [8]. Now managed by the Medicines and Healthcare products Regulatory Agency (MHRA), the YCS serves as an early warning system for ADRs not observed during clinical trials, relying on reports from healthcare professionals, patients and caregivers. However, spontaneous reporting systems like the YCS are often limited by under-reporting and data inconsistencies [9].

The European Molecular Biology Laboratory (EMBL) have advanced drug research by developing rich, structured databases using natural language processing and text-mining to extract insights from the medical and scientific literature, patents and assays [10]. Key resources include STITCH (Search Tool for Interactions of Chemicals), SIDER (Side Effects Resource) and ChEMBL (The European Molecular Biology Laboratory’s bioactivity database) [9–12] have grown significantly in scope and quality over the past decade. When integrating this data with insights from the YCS on ADRs, previously unrecognised patterns can be revealed. Machine learning (ML) can uncover these latent relationships, enable improved ADR prediction and enhance drug safety.

While ML offers promise in healthcare, its clinical adoption depends on accuracy, interpretability and reliability [13–14]. Boland et al. [15] highlighted diverse methods and datasets used to enhance ADR prediction, while the work of Ietswaart et al. [16] has been particularly influential in the design of this study’s approach.

The research aims to develop a predictive framework that not only identifies ADRs from drug-target interaction data but also provides clear, interpretable insights into why those predictions are made. By uncovering the relationships between drugs, targets and ADRs, this approach contributes to a deeper pharmacological understanding, moving beyond prediction to reveal mechanisms that could inform safer drug design and use.

## 2 Materials and methods

### 2.1 Drug interaction data

Drug-target interaction data was obtained from STITCH v5.0 (accessed [16^th^ Jan 2025]) [17]. Data files were downloaded for human interactions and chemical identifiers, providing the information for interactions between drugs and human targets. Each interaction is associated with a confidence score ranging from 0 (no confidence) to 1 (high confidence); interactions with missing data or no supporting evidence were conservatively assigned a score of 0.

The dataset was filtered to include only the drugs found in the YCS (see section 2.2). STITCH assigns confidence scores to interactions through computational prediction based on five sources: Genomic context predictions, high-throughput lab experiments, (conserved) co-expression, automated text-mining, and curated databases [17]. It also provides ‘transfer scores’ for interactions derived from homologous or orthologous data from other organisms. Although the inclusion of these transfer scores substantially increased the dataset size, improving prediction potential, it relied on the assumption that the transferred interactions were accurate. STITCH groups scores into brackets, with those below 0.4 considered low confidence [17]. A threshold of 0.4 was thus applied to retain only medium and high-confidence interactions, more likely to reflect true pharmacological processes. This threshold balanced data quality and quantity; while stricter filtering might have further reduced potential false positives, it risked omitting interactions that could be informative. Low confidence scores were then assigned a score of 0. Filtering yielded 1,248 drugs and 6,573 targets identified with interactions with values between 0 and 1.

### 2.2 ADR Data

#### Structuring and selecting data.

ADR data was obtained from the YCS (accessed [3^rd^ Jan 2025]) [18], which compiles spontaneous reports of suspected ADRs in the UK. The dataset contained 3,097,997 records, structured using the Medical Dictionary for Regulatory Activities (MedDRA) hierarchical system [19]. MedDRA is an internationally recognised standard for classifying ADRs structured across five hierarchical levels:

- System Organ Classes (SOC) – 27 terms
- High-Level Group Terms (HLGT) – 337
- High-Level Terms (HLT) – 1,737
- Preferred Terms (PT) – 25,592
- Lowest Level Terms (LLT) – 85,668

This study focused on the SOC level due to known limitations in spontaneous reporting systems, including under-reporting and related data inconsistencies [9], which could skew the results at finer hierarchical levels. Aggregation at the SOC level reduces these issues and has been shown to improve the robustness of pharmacovigilance methods, reduce bias, and enhance the reliability of the analysis [20–21].

Certain SOC categories were excluded for lack of relevance to pharmacological action:

- General disorders and administration site conditions.
- Injury, poisoning, and procedural complications.
- Investigations.
- Product issues.
- Social circumstances.
- Surgical and medical procedures.

This aligns with methodologies used in prior studies, excluding non-pharmacological SOC categories and focussing on ADRs potentially associated to a pharmacological action [22].

#### Programmatic retrieval of data.

Data extraction involved computational techniques to automate the retrieval and preparation of data. The YCS website contains drug-specific links identified in the HTML structure, following a consistent URL pattern, allowing programmatic generation of direct links to associated zipped data files. Using Python’s requests library, data for all 2,507 drugs on the YCS website [18] was downloaded and consolidated into a master file. Of these 2,507 drugs, 1,248 overlapped with the STITCH dataset and were included in the study. This disparity stemmed from the lack of availability of drug identifier keys within each database.

#### Signal detection via disproportionality analysis.

Disproportionality analysis was conducted to identify statistically significant ADR signals, a validated quantitative method used in pharmacovigilance [23]. Two metrics were applied: the reporting odds ratio (ROR) and proportional reporting ratio (PRR), further details are located in S1 Appendix Additional Methods. The threshold of >2 was used against both metrics to signify statistical significance [23–25]. While confidence intervals (e.g., 95% confidence interval) are often used to indicate statistical significance, differences in the data meant this could not be applied consistently. Therefore, in the absence of a universal benchmark, the > 2 threshold was selected as a practical benchmark for comparability across datasets. For classification, the following was performed:

- ADRs below the statistical significance threshold were assigned a value of 0.
- ADRs above the statistical significance threshold were assigned a value of 1.
- Missing data was assigned a value of 0.

This methodological approach yielded binary classification labels to map interaction data to statistically significantly classified ADRs. This method accounts for frequency, not severity, of reported events, which is influential in pharmacological prediction. However, pharmacovigilance efforts may process this differently.

#### Comparative Analysis of Clinical and Real-World ADR Data.

SIDER v4.1 [26] was used to compare ADR signals from both spontaneous reporting systems and clinical trials. SIDER contains ADR data from clinical trials and drug labels, offering insights into information from controlled clinical environments [11]. Comparing clinical trial (CT) data and spontaneous reporting systems highlighted biases and differences between controlled CT and real-world evidence (RWE). This comparison further supports why controlled clinical trials may miss certain ADR signals that spontaneous reports capture, validating the use of systems like the YCS for identifying additional ADRs post-marketing.

To facilitate this comparison, we used the ADR percentages as a numerical value and subjected them to disproportionality analysis to extract the significant ADRs. The CT and RWE datasets were analysed using the same thresholds for ROR and PRR, enabling direct comparison of significant drug-ADR pairs.

**Fig 1:**
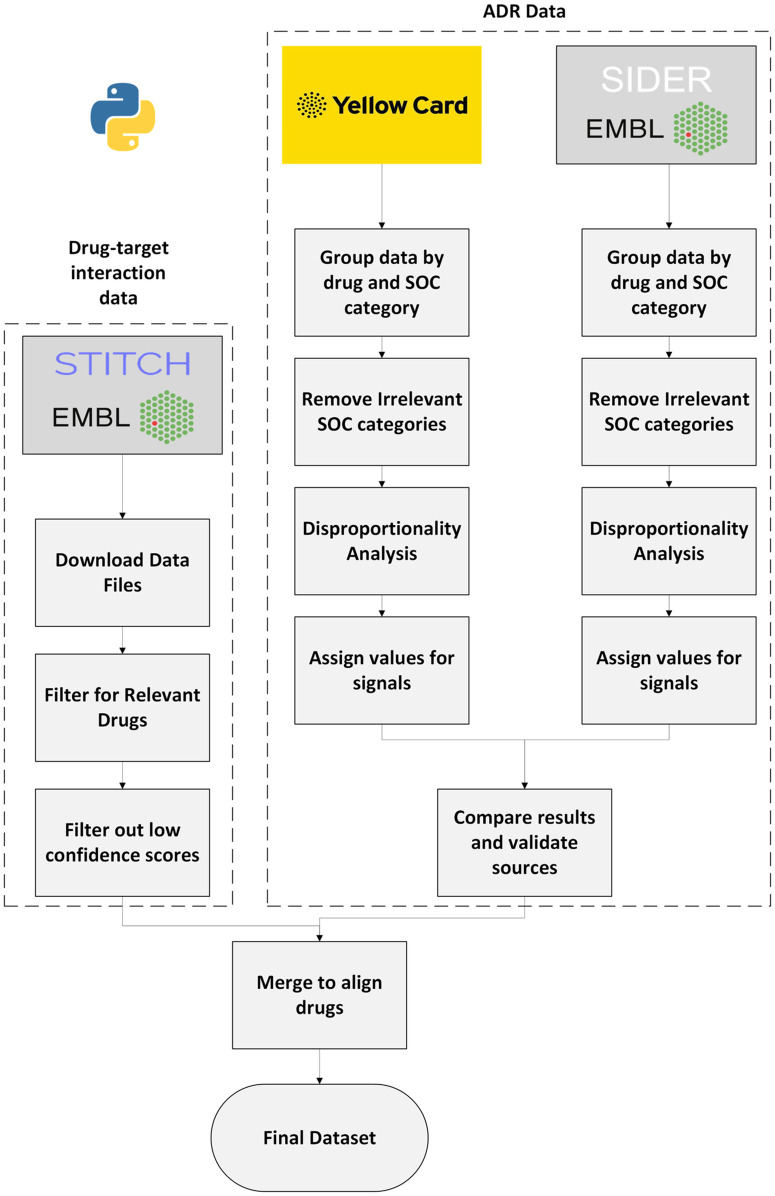
Flowchart of database collection methods.

The Jaccard index was used to compare overlaps between significant ADR signals, which is further outlined in S1 Appendix Additional Methods. The Jaccard index accounts for only the significant ADR signals and compares the overlap from both datasets to the total significant signal proportion. This allows a percentage similarity score to be generated. The overall workflow for database creation is shown in Fig 1.

### 2.3 Machine Learning

#### Justification for random forest models.

Random Forests were selected as the primary modelling approach due to their strong balance between predictive power and interpretability [27], crucial for tracing ADR signals back to pharmacological predictors. Unlike deep-learning models, which operate as ‘black boxes’ with limited transparency, Random Forests offer feature importance that supports explainability in clinical settings, fostering confidence and enabling informed decision-making [28–29]. They also perform well on high-dimensional and noisy datasets, avoiding overfitting through ensemble learning [30–31]. Compared to other interpretable models, such as decision trees or logistic regression, Random Forest models provide superior performance, capturing complex, non-linear relationships between features without sacrificing transparency [32–33]. Their successful application in medical tasks further supports their relevance and reliability in this context [34]. These qualities make Random Forests the most appropriate choice for developing an interpretable yet effective framework.

#### Model development and evaluation.

Random Forest models were used to identify patterns between drug-target interactions and ADRs using the scikit-learn (v1.6.1) package [35]. A binary classification approach was adopted to detect if an ADR was significant or not, based on drug-target interaction data. A separate Random Forest classifier was trained for each SOC category to simplify the models and allow for key interactions to be identified for specific ADR categories using feature importance.

Model performance was evaluated on a proportion of the dataset categorised for testing using the receiver operating characteristic area under the curve (ROC AUC), chosen for its robustness in evaluating model performance in the presence of class imbalance [36]. To provide a comprehensive evaluation, additional metrics, including precision, recall, accuracy, F1 score, and Matthews Correlation Coefficient (MCC), were calculated, offering insights into various aspects of model performance. (See S1 Appendix Additional Methods)

#### Handling class imbalance: SMOTE + Tomek links.

Class imbalance is common in ADR prediction, with significant ADRs representing the minority class, especially deeper in the MedDRA hierarchy [37–38]. To address this, we applied Synthetic Minority Over-sampling Technique (SMOTE) combined with Tomek links, an under-sampling technique, to better balance the data [39–40]. SMOTE generates synthetic data for the minority class while Tomek links reduce borderline examples of the majority class. This hybrid approach aimed to increase the number of meaningful positive examples while reducing noise and redundancy from the majority class. Despite concerns about synthetic data remaining valid, this approach improved classification performance, suggesting utility in pharmacovigilance applications. Still, further refinement may enhance synthetic data fidelity and generalisability.

#### Bayesian optimisation of hyperparameters.

Bayesian optimisation is a probabilistic model-based method for hyperparameter tuning. It efficiently explores the search space when evaluation is computationally expensive [41]. It balances exploring new configurations and exploiting areas known to yield high performance, which is promising compared to traditional grid or random search methods. In this study, Bayesian optimisation was applied to fine-tune these hyperparameters of the models [35]:

- Number of trees (n_estimators)
- Minimum number of samples required to split an internal node (min_samples_split)
- Minimum number of samples required to be at a leaf node (min_samples_leaf)
- Number of features to consider when looking for the best split (max_features)
- Weights associated with classes (class_weight).

In total, 100 trials were conducted for each model, facilitating efficient hyperparameter exploration and contributing to improved model performance.

#### Comparison of random forests against decision trees.

To ensure Random Forests offered genuine performance benefits over simpler interpretable models, their results were compared to decision trees. While decision trees provide maximum interpretability via clear rule-based splits, they are more prone to overfitting and limited in capturing complex interactions. In contrast, Random Forests deliver superior predictive performance through ensemble averaging, reducing variance and improving generalisability [32]. This comparison reaffirms the earlier justification for selecting Random Forests, offering a strong trade-off between interpretability and performance, crucial for reliable prediction in clinical contexts.

#### Model interpretation using feature importance and cross-validation with DisGeNET.

Random Forests offer interpretability through feature importance scores, quantifying the contribution of individual features to the models’ predictions [42]. These scores offer insights into the targets that most significantly influence the predictions of significant ADRs.

**Fig 2:**
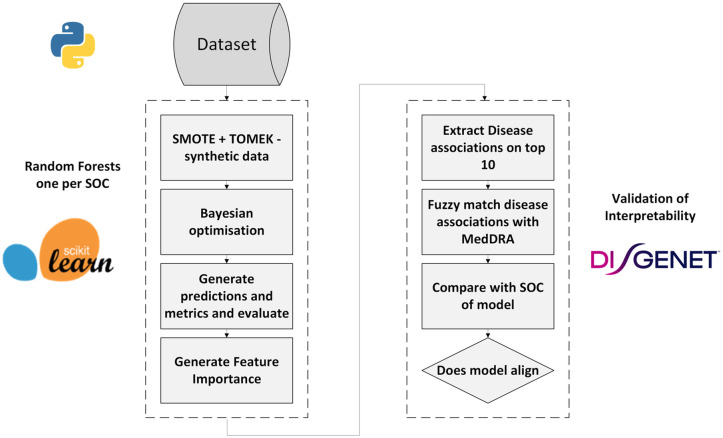
Flowchart of ML methods excluding comparison with Decision trees.

To validate the interpretability of models, the top 10 targets identified by the Random Forests were cross-referenced with DisGeNET, a comprehensive repository of target-disease relationships [43]. Then, a fuzzy matching algorithm was implemented using the “fuzzywuzzy” Python package [44] to map the extracted target-disease associations to their most similar MedDRA classifications. This allowed a direct comparison between the SOC category of each model and the SOC category of associated diseases. Models where target-disease associations aligned with the original SOC category were considered effective, ensuring meaningful and interpretable outputs. The overall workflow of model creation and validation is depicted in Fig 2.

#### Comparison to drug interactions.

In the final step, a comparison was made back to the drugs extracted from the YCS. The interactions found to be important by the Random Forest model were cross referenced to the drugs they interact with and then compared to their Anatomical Therapeutic Chemical (ATC) classification [45] to gain an understanding in a pharmaceutical context within drug groups. This provided a secondary validation technique to understand the capabilities of this method relating back to drugs that have a higher chance of being associated with side-effects from interacting with common pharmacological targets.

#### Ethics statement.

This study involved only publicly available data and did not include any private or identifiable patient information; therefore, no ethical approval was required.

## 3 Results

### 3.1 Comparative analysis of clinical and real-world ADR data

**Fig 3:**
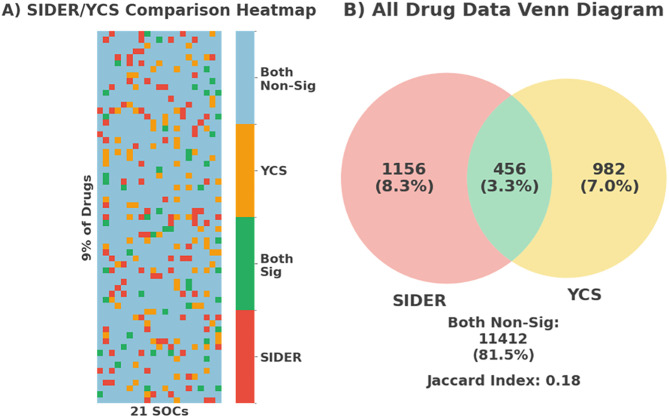
A) Heatmap comparison of Drug-ADR associations between the SIDER database (clinical trials) and the YCS (real-world reports) datasets across MedDRA SOC categories. B) Venn Diagram representing all drug data proportions of each category in heatmap.

The comparison of RWE data to the SIDER database yielded the following results, visualised in a heatmap shown in Fig 3A. To enhance interpretability 9% of the drugs are highlighted. The X-axis shows each SOC model in alphabetical order. Fig 3B represents the distribution of data. Most ADR categories in both datasets were insignificant, as expected. However, of the noteworthy results, only 3.3% of the significant signals overlapped. In contrast, 7.0% were unique to the YCS and only 8.3% were only found in SIDER. The Jaccard index revealed that approximately 17.6% of significant signals are shared between clinical trials and real-world reports. This low degree of overlap suggests that a large majority (82.4%) of significant findings are unique to one of the datasets.

### 3.2 Evaluation of model predictions

#### Overview of model predictions.

**Fig 4:**
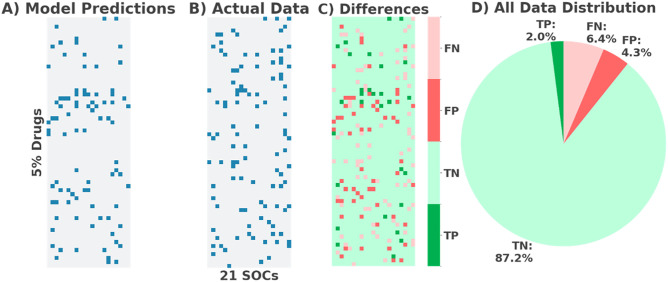
A) Heatmap of model predictions. B) Heatmap of actual data. C) Heatmap of similarities and differences in model predictions and actual data. D) Pie chart representing distribution of predictions on all drug data for drug-ADR associations across MedDRA SOC categories.

All results in Fig 4 relate to the test data. Model predictions are shown Fig 4A, the actual data is shown in Fig 4B (blue – significant, white – insignificant). Fig 4C Shows a comparison heatmap and Fig 4D represents the distribution of prediction results for all data. The model predictions aligning with the test dataset are categorised as correct and represented in green and the unaligned predictions, or incorrect ones in red. To enhance interpretability Fig 4A-4C presents 5% of the total drugs in the dataset (25% of the test data). The X-axis shows each SOC model in alphabetical order. An overview of the outcomes is:

- 87.2% true negatives (TN) – correct predictions of non-significant ADRs.
- 2.0% true positives (TP) – correct predictions of significant ADRs.
- 6.4% false negatives (FN) – incorrect predictions of significant ADRs.
- 4.3% false positives (FP) – incorrect predictions of non-significant ADRs.

This reflects strong class imbalance, with non-significant ADRs dominating. Full prediction outcomes are detailed in the S1 Appendix Tables, showing further variation across models.

#### Model performance metrics summary.

**Fig 5:**
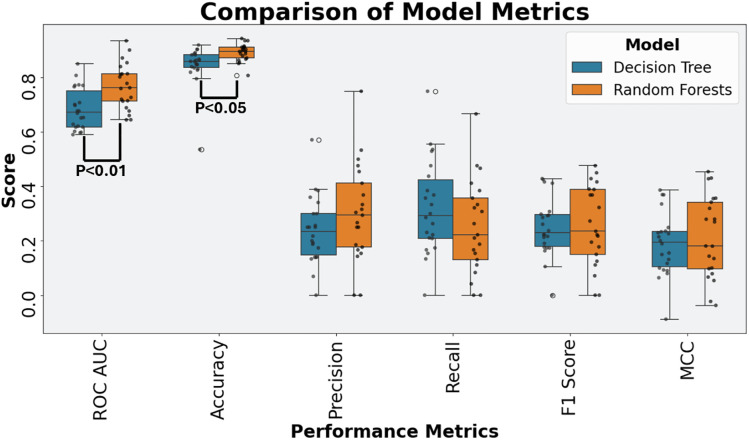
Performance metrics of the 21 SOC models for Decision Trees and Random Forests with significant differences indicated in ROC AUC and accuracy.

Model performance was evaluated using multiple metrics, including ROC AUC, accuracy, precision, recall, F1-score, and MCC. As noted in section 2.3, models were optimised based on their ROC AUC values during Bayesian hyperparameter tuning. Decision tree and Random Forests results are visualised in Fig 5. Random Forests significantly outperform decision trees in ROC AUC and accuracy, t-test results can be found in S1 Appendix Tables. Complete metric values are provided in S1 Appendix Tables. A summary of Random Forest performance is provided below:

- ROC AUC: 0.64–0.94
- Accuracy: 0.81–0.94
- Precision: 0.00–0.75
- Recall: 0.00–0.67
- F1-score: 0.00–0.48
- MCC: −0.04–0.45

The metric highlights variation across the 21 SOC models. Some models demonstrate strong classification ability, particularly with high ROC AUC and accuracy. Accuracy remained consistently high in the context of a dataset where non-significant ADRs were more prevalent. However, other metrics such as precision, recall, F1-score, and MCC showed lower results, indicating challenges in correct predictions.

### 3.3 Interpretable Insights from the Top-Performing Model

#### Identifying influential targets in ADR prediction.

**Fig 6:**
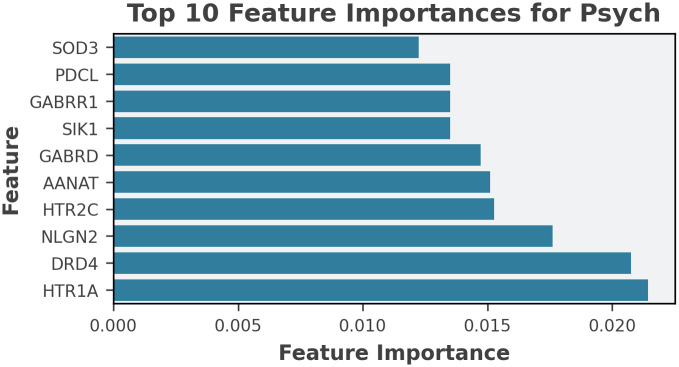
Random Forest feature importance results of the top 10 targets for the Psychiatric Disorder model.

To understand the interpretability of the method, we performed a feature importance analysis using a case study of psychiatric disorders. This model was the highest performing regarding the metrics and was expected to provide the most reliable interpretation. Fig 6 presents a bar chart of the top 10 features, most commonly contributing to the models’ positive predictions. These values indicate the relative influence of each target as an input to the model.

#### Validating influential targets using DisGeNET.

To validate the interpretability of the model, we cross-referenced the targets identified in the feature importance analysis against the DisGeNET database to identify established target-disease associations. DisGeNET provides documented associations, which we mapped to the MedDRA hierarchy system, categorising them by their respective SOC categories.

**Fig 7:**
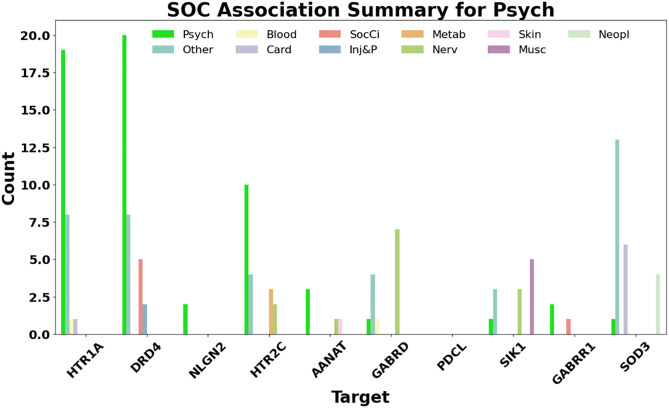
Summary of SOC category associations for the top 10 targets based on feature importance, sourced from DisGeNET.

Fig 7 Summarises these associations by presenting the count of SOC categories linked to each target’s disease associations. For clarity, the figure highlights the bar corresponding to the psychiatric disorders in green alongside the next 2 most common SOC categories. All remaining categories have been grouped under “other”.

Among the top 10 targets, 9 have known associations with psychiatric disorders. One target, PDCL, does not contain any data on DisGeNET and is shown with no known associations.

### 3.4 Drug Relations of Top Targets

**Fig 8:**
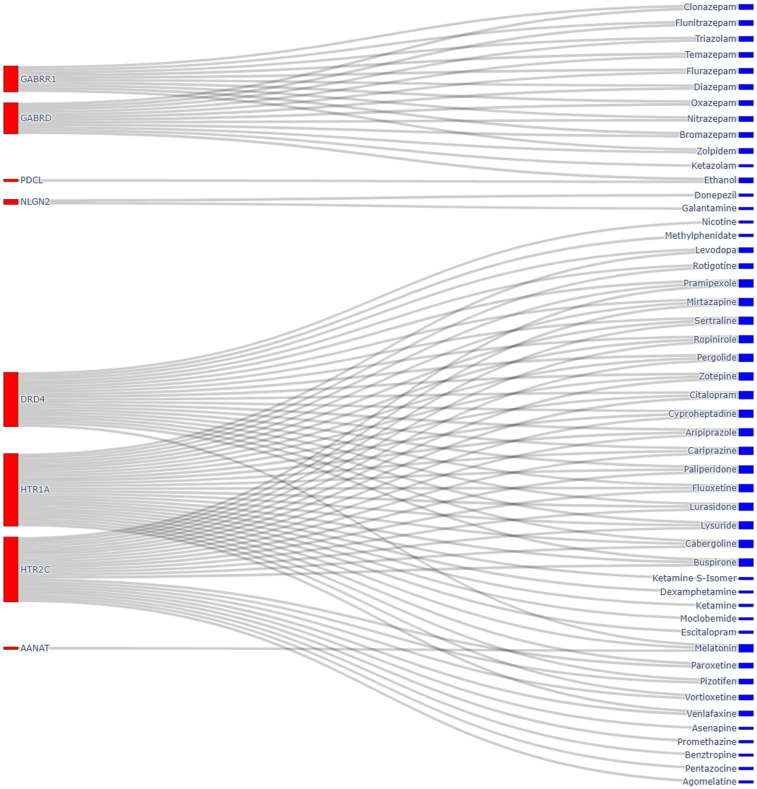
Target Drug Relations linked to the top 10 targets based on feature importance.

The Top 10 targets identified by the models were cross-referenced against the drugs with which they interact. These results are shown in Fig 8 where the lines represent interactions, the size of the colour bar next to the text relates to count, the larger the bar, the more interactions. Of the drugs listed, 46 of 49 belong to the ATC Classification prefix N for Nervous System, as seen in S1 Appendix Tables. Showing a strong correlation between this category of ATC group and psychiatric disorders.

## 4 Discussion

### 4.1 Comparative Analysis of Clinical and Real-World ADR Data

The comparative analysis between the YCS and SIDER databases reveals a striking divergence in ADR signals. Only 3.3% of significant ADRs cross-over, compared to 7.0% and 8.3% uniquely found in the YCS and SIDER. The low Jaccard index (17.6%) further reinforces the minimal overlap between clinical trials and real-world datasets. This highlights how the sources capture different ADR profiles, consistent with prior studies’ findings [46–48]. These differences stem from methodologies, populations, and data collection contexts, which are explored below.

#### Limitations of spontaneous ADR reporting.

The YCS is a spontaneous reporting system, providing real-world post-marketing drug safety reports. However, these systems are limited, which can bias the ADR profile of drugs. One major issue is under-reporting. It is estimated that less than 10% of all ADRs are reported [49], creating significant gaps in pharmacovigilance data. This under-reporting is influenced by multiple factors, including lack of awareness, clinical time constraints, or the perception that an event is not serious or not drug-related [50].

Reporting bias complicates data reliability. Healthcare professionals are more likely to report severe or unexpected ADRs and not report well-known but potentially impactful ADRs [49, 51]. Meanwhile, patient reports may overemphasise subjective symptoms or be influenced by personal expectations and media narratives. For instance, Van Hunsel et al. [52] found media coverage of statin-related ADRs significantly increased patient reports, many emotional in tone and difficult to validate clinically. This highlights the susceptibility of these systems to “simulated reporting,” where attention-driven surges may distort the data.

Data completeness and quality are also issues. Reports often lack essential clinical details, like dosage, concurrent medications or patient history, making report assessment challenging [15]. This information could limit the validity of data, meaning assumptions of correctness must be made.

#### Constraints of clinical trial-based ADR detection.

Detecting ADRs in clinical trials is essential for safety evaluation, yet subject to biases that limit its reliability. One key issue is capturing and reporting ADRs. Clinical trials are conducted in highly controlled environments with strict inclusion and exclusion criteria, often excluding comorbidities, children, older adults, pregnant women, or those taking multiple medications [53–55]. This selection bias results in study populations that do not neccessarily reflect the broader therapeutic usage in the public [56].

The duration and size of trials are often insufficient to detect rare or long-term ADRs. Pre-marketing trials typically involve hundreds to low thousands of participants and span weeks to months. Many ADRs occur with prolonged exposure or are rare, requiring larger populations for detection [57–58]. This limitation is particularly problematic for low-frequency but high-impact events such as liver toxicity, cardiovascular risks, or neuropsychiatric problems.

Under-reporting bias also plays a key role. Symptoms may be perceived as unrelated to drugs, particularly if they resemble pre-existing conditions. As Corrigan argues [57], trial framing often leads to ADRs being under-detected or misclassified. Ioannidis [59], further critiques the design and utility of clinical studies, suggesting a significant proportion have limited practical application. Thus, clinical trials may not offer a complete picture of a drug’s ADR profile, underscoring the value of spontaneous reporting systems.

#### Structural and contextual differences in ADR data.

While both datasets capture ADR data, they differ fundamentally in structure and information. The YCS provides raw reports across various SOC categories, whereas SIDER offers percentage-based probability data. Methodological distinctions, including the disproportionality analysis used for comparison, further contribute to disparities in the results.

Broader contextual factors, such as inherent reporting biases, further explain these discrepancies. Clinical trials may under-represent real-world populations or overlook long-term side effects. While spontaneous reporting systems such as the YCS, despite under-reporting issues, capture a wider, more diverse population. Given these nuances, the YCS emerges as a valuable resource for subsequent analysis. Its inclusivity and real-world relevance enhance its capacity to detect otherwise missed safety signals, justifying its use as the representative dataset for identifying ADR signals.

### 4.2 Evaluation of Model Predictions

#### Impact of data maturity on prediction performance.

The predictive performance of the Random Forests indicates moderate but uneven levels of effectiveness in identifying ADRs from drug-target interaction data. While some models achieved strong classification metrics (ROC AUC and accuracy values up to 0.94), others performed poorly, with low precision, recall, F1-scores, and MCC values. A high proportion of true negatives (87.2%) and a low false positive rate (4.3%) suggest reliable identification of non-significant ADR signals. However, the low true positive rate (2.1%) and higher false negative rate (6.4%) reflect persistent challenges in detecting significant ADRs, driven by class imbalance and variable signal quality across SOCs. These results show promise for certain SOCs but highlight the need for further refinement. The variation in model outcomes highlights the importance of SOC-specific adaptation to improve sensitivity and clinical relevance in ADR prediction. Comparison with decision trees validates that Random Forests are better suited as they outperformed them significantly in key metrics of ROC AUC and accuracy, and trends would suggest this in the other metrics studied.

#### Source bias and its effect on model learning.

A contributor to the performance variation across SOCs is disparity in data quality and quantity from the primary sources (YCS and STITCH). STITCH reflects research trends and publication biases, which may lead to certain drug-ADR associations indirectly being well-represented while others remain under-represented. The YCS contains bias from selective reporting and under-reporting, contributing to differences in data quality and quantity. Models trained on well-represented categories may benefit from cleaner, more informative features, and under-represented categories face noisier, lower-quality data. This unevenness affects the model’s ability to learn meaningful patterns.

This aligns with perspectives in machine learning research. Halevy et al. [60] noted, “more data usually beats a clever algorithm”, emphasising the value of data richness. More recently, Simon et al. [61], reinforced that larger, higher-quality datasets can improve generalisation and model performance.

#### Evaluation of hyperparameter optimisation and model tuning.

Bayesian hyperparameter tuning facilitated efficient identification of near-optimal configurations. Known for its capability to balance exploration and exploitation in complex search spaces [62], this method proved advantageous here. While the global optimum may not have been reached, the consistency of results across iterations indicates sufficient optimisation, supporting the viability of the approach.

#### Metric selection in the context of class imbalance.

Although many models demonstrated high accuracy, this metric poorly reflected true performance due to class imbalance. In early iterations, some models surpassed 98% accuracy by predicting all cases as non-significant. These models, despite seeming effective, failed to identify true positives, rendering them ineffective for ADR prediction. Moreover, under severe class imbalance, accuracy and even ROC AUC can overstate performance, whereas precision/recall/F1 and MCC were lower and showed little separation between models. This limitation motivated reporting ROC AUC alongside class-sensitive metrics and suggests future exploration of cost-sensitive learning or alternative loss functions in the training process. Penalising false negatives more heavily could prioritise the detection of significant ADR signals, resulting in more clinically valuable models.

### 4.3 Interpretable Insights from the Top-Performing Model

#### Identifying influential targets in ADR prediction.

An advantage of interpretable models is their ability to extract meaningful insights, with Random Forests, this is through feature importance. Feature importance provides insight into how often and how influential each feature was when splitting the data during training, specifically for significantly predicted ADRs. By isolating contributions from positive predictions, we identified targets most consistently linked to positive ADR signals. Although individual scores were small due to high dimensionality, recurring influential features emerged. This targeted analysis offers valuable direction for understanding ADR mechanisms and supports future pharmacological investigation and risk profiling.

#### Validating influential targets using DisGeNET.

The alignment between the model’s most influential features and known target-disease associations highlights its interpretability and pharmacological relevance. Notably, 9 out of the top 10 targets identified by the best-performing model matched known psychiatric disorder associations in DisGeNET, reinforcing confidence in the model’s capability to identify true contributors to ADR signals. This suggests models are not capturing noise but instead are identifying meaningful patterns within the data.

The remaining gene, PDCL, currently undocumented in DisGeNET, could also represent a novel target linked to the nervous system effects of alcohol (ethanol) [63]. This highlights the model’s potential to suggest new avenues for experimental validation. These findings demonstrate the broader utility of this framework, not only as a predictive tool but also as a platform for discovery. In pharmacovigilance, where interpretability is vital, the ability to explain known ADR mechanisms and propose new ones is a significant advantage.

### 4.4 Drug Relations from Top Targets

An outsize proportion of the drugs linked to the targets belong to the ATC prefix N for nervous system. This suggests that drugs linked to the nervous system ATC classification have a high likelihood of interacting with common targets related to psychiatric side effects. However, it is important to note that this observation may be confounded, as psychiatric conditions may worsen during treatments with psychiatric drugs, which could inflate apparent associations between these drugs and psychiatric ADRs. Nonetheless, other literature that includes large-scale pharmacovigilance analyses showing that nervous system drugs are consistently overrepresented in adverse event reporting. Aagaard and Hansen [64] found that nearly half of consumer-reported ADRs for ATC group N medications were serious, with psychiatric and nervous system disorders among the most common outcomes. Similarly, Wu et al. [65] identified nervous system drugs as carrying one of the highest burdens of serious ADRs, while further analyses of psychotropic agents [66] reinforced their strong association with psychiatric side effects. Collectively, these findings align with our results, supporting the observation that nervous system drugs are disproportionately linked to targets associated with psychiatric adverse reactions.

### 4.5 Advancing ADR Predictive Models: Challenges and Future Directions

#### Constraints in model development and optimisation.

Model creation and optimisation were constrained by computational limitations, making processes like hyperparameter tuning time intensive. Greater computing power could streamline this workflow through parallelisation, reducing reliance on sequential execution. Although Bayesian optimisation performed well, increased resources might uncover better-performing configurations. Addressing this bottleneck could improve the performance and scalability of ADR predictive frameworks.

#### Challenges in data loss due to inconsistent drug descriptors.

A key challenge was inconsistency in drug descriptors. Many drugs were lost during mapping between datasets due to missing identifiers like Simplified Molecular Input Line Entry System (SMILES) keys, which are critical for aligning chemical structures. Further complications arose from differing naming conventions, drugs were often referred to by synonyms or brand names, leading to incorrect representations and data loss. This caused the reduction from 2,507 drugs in the YCS to 1,248 drugs in the final dataset. Addressing this issue requires the standardisation of chemical identifiers, ensuring accurate mapping and improving data integration.

#### Improving models with global pharmacovigilance data.

Integrating additional spontaneous reporting systems, like FAERS (FDA Adverse Event Reporting System) and JADER (Japanese Adverse Drug Event Report) databases [67], would increase data volume and diversity, potentially leading to more reliable and generalisable results. Integrating such datasets could counteract under-reporting and inconsistencies, creating a stronger foundation for ADR prediction.

#### Strengthening performance against imbalanced data.

Addressing data imbalance remains critical, especially for detecting rare yet significant ADR signals. Imbalance creates issues in all machine learning tasks [68]. This challenge is equally evident in this study. Beyond metric selection and loss weighting, synthetic data generation may improve ADR prediction. This study applied SMOTE oversampling to enrich significant ADR signals with Tomek links to reduce noise, yielding modest gains in performance. However, future models may benefit from more advanced generative methods, like autoencoders or Generative Adversarial Networks (GANs) [69], which may produce higher quantities of realistic samples. GANs were not employed in this study due to their complexity and limited interpretability but remain a promising direction for future research.

#### Enhancing ADR insight through deeper MedDRA hierarchies.

Improvements in model capability and data quality could allow exploration at deeper MedDRA levels, for example the preferred term (PT) level, offering more precise insights into specific drug-target-ADR relationships. Greater granularity could uncover associations not visible at higher levels of classification. However, increased data imbalance at these levels limits the available possibilities. Overcoming this challenge could enhance ADR prediction, developing a deeper understanding of drug safety.

#### Using interpretable models for enhanced drug safety.

This methodology provides a promising framework for linking targets, drugs, and ADRs using interpretable machine learning. These models provide valuable insights missed by ‘black box’ approaches like neural networks. By identifying pharmacological interactions contributing to ADR signals, the approach enables the validation of potential mechanisms in operation. Thus, supporting the development of strategies to mitigate and prevent associated adverse outcomes. Such insights may improve drug safety and allow for the improvement of personalised medicine, tailoring treatment to individuals’ genetic profiles based on predicted risk.

Furthermore, this methodology could guide drug discovery. Identifying drug-target interactions linked to ADRs could help improve the safety profile of future drugs. As drug-target identification networks evolve, this approach could serve as a feedback mechanism, refining early-stage decisions. Integrating this methodology with drug-target interaction prediction models could enable the early identification of ADR associations, supporting risk mitigation during drug development. This integration holds potential for creating a more proactive and precise drug design process, though further research is required to address pharmacological complexities and genetic variability.

## 5 Conclusions

The study presents an interpretable machine learning framework that leverages drug-target interaction data to predict ADRs and provide pharmacologically meaningful insights. Using Random Forests trained on curated data from STITCH and the YCS, the framework identifies associations between drugs, targets and ADRs across 21 MedDRA SOCs. Model interpretability was reinforced through external validation against DisGeNET, revealing alignment between predicted targets and known disease associations, thereby supporting both the pharmacological plausibility and utility of the framework.

Variability in model performance across ADR categories reflected underlying disparities in data quantity, quality, and class imbalance. While these limitations constrained predictions in under-represented categories, the approach, including Bayesian optimisation and SMOTE with Tomek links, demonstrated strong potential in extracting clinically relevant signals from noisy and imbalanced data. Identifying influential targets adds significant value beyond accuracy, reinforcing the framework’s relevance for pharmacological insight and early-stage safety screening. Additionally, the association between nervous system drugs and psychiatric side effects further underscores the framework’s ability to capture clinically meaningful patterns, reinforcing its potential utility in identifying higher-risk therapeutic areas.

Future work should focus on integrating broader pharmacovigilance sources to address under-reporting and enrich signal diversity. Additionally, more advanced generative techniques and exploration of deeper MedDRA hierarchies may enhance understanding of specific ADR pathways. With these enhancements, the framework could contribute meaningfully to safer drug development and usage and a more proactive pharmacovigilance landscape.

## Supporting information

S1 Appendix Additional methods (Disproportionality Analysis, Jaccard Index, Performance Metrics). Tables (Table 1 – Model Prediction Values, Table 2 – Random Forest Metrics Results, Table 3 – Decision Tree Model Metrics, Table 4 -t-test values for Decision Tree and Random Forest comparison of model metrics, and Table 5 – Drugs of Interest ATC codes.
(PDF)

## References

[1] Osanlou R, Walker L, Hughes DA, Burnside G, Pirmohamed M. Adverse drug reactions, multimorbidity and polypharmacy: a prospective analysis of 1 month of medical admissions. BMJ Open. 2022;12(7):e055551. doi: 10.1136/bmjopen-2021-055551 35788071 PMC9255409

[2] Rawlins MD, Thompson JW. Mechanisms of adverse drug reactions. Drugs. 1977;14:393–408.

[3] Moncrieff J, Cooper RE, Stockmann T, Amendola S, Hengartner MP, Horowitz MA. The serotonin theory of depression: a systematic umbrella review of the evidence. Mol Psychiatry. 2023;28(8):3243–56. doi: 10.1038/s41380-022-01661-0 35854107 PMC10618090

[4] Tozer TN, Rowland M. Introduction to pharmacokinetics and pharmacodynamics: the quantitative basis of drug therapy. Philadelphia: Lippincott Williams & Wilkins. 2006.

[5] Pirmohamed M. Mechanisms of adverse drug reactions. In: Mann’s pharmacovigilance. John Wiley & Sons, Ltd; 463–87.

[6] WHO EDM 2004.8. https://iris.who.int/bitstream/handle/10665/68782/WHO_EDM_2004.8.pdf

[7] Guidance_on_adverse_drug_reactions. Accessed 28 November 2024. https://assets.publishing.service.gov.uk/government/uploads/system/uploads/attachment_data/file/949130/Guidance_on_adverse_drug_reactions.pdf

[8] The Yellow Card scheme: guidance for healthcare professionals, patients and the public. 2021. Accessed 2025 February 21. https://www.gov.uk/guidance/the-yellow-card-scheme-guidance-for-healthcare-professionals

[9] Avery AJ, Anderson C, Bond CM, Fortnum H, Gifford A, Hannaford PC, et al. Evaluation of patient reporting of adverse drug reactions to the UK “Yellow Card Scheme”: literature review, descriptive and qualitative analyses, and questionnaire surveys. Health Technol Assess. 2011;15(20):1–234. doi: 10.3310/hta15200 21545758

[10] Szklarczyk D, Santos A, von Mering C, Jensen LJ, Bork P, Kuhn M. STITCH 5: augmenting protein-chemical interaction networks with tissue and affinity data. Nucleic Acids Res. 2016;44(D1):D380-4. doi: 10.1093/nar/gkv1277 26590256 PMC4702904

[11] Kuhn M, Letunic I, Jensen LJ, Bork P. The SIDER database of drugs and side effects. Nucleic Acids Res. 2016;44(D1):D1075-9. doi: 10.1093/nar/gkv1075 26481350 PMC4702794

[12] Zdrazil B, Felix E, Hunter F, Manners EJ, Blackshaw J, Corbett S, et al. The ChEMBL Database in 2023: a drug discovery platform spanning multiple bioactivity data types and time periods. Nucleic Acids Res. 2024;52(D1):D1180–92. doi: 10.1093/nar/gkad1004 37933841 PMC10767899

[13] Abdullah TAA, Zahid MSM, Ali W. A review of interpretable ML in healthcare: taxonomy, applications, challenges, and future directions. Symmetry. 2021;13(12):2439. doi: 10.3390/sym13122439

[14] Ghassemi M, Naumann T, Schulam P, Beam AL, Chen IY, Ranganath R. A review of challenges and opportunities in machine learning for health. AMIA Jt Summits Transl Sci Proc. 2020;2020:191–200. 32477638 PMC7233077

[15] Boland MR, Jacunski A, Lorberbaum T. Systems biology approaches for identifying adverse drug reactions and elucidating their underlying biological mechanisms. Wiley Interdiscip Rev Syst Biol Med. 2016;8:104–22. 26559926 10.1002/wsbm.1323 PMC4760887

[16] Ietswaart R, Arat S, Chen AX, Farahmand S, Kim B, DuMouchel W, et al. Machine learning guided association of adverse drug reactions with in vitro target-based pharmacology. EBioMedicine. 2020;57:102837. doi: 10.1016/j.ebiom.2020.102837 32565027 PMC7379147

[17] STITCH: chemical association networks. Accessed 2025 May 1. http://stitch.embl.de/

[18] Yellow Card | Making medicines and medical devices safer. Accessed 2025 April 10. https://yellowcard.mhra.gov.uk/

[19] MSSO updates. MedDRA. Accessed 2025 April 11. https://www.meddra.org/

[20] Koutkias VG, Jaulent M-C. Computational approaches for pharmacovigilance signal detection: toward integrated and semantically-enriched frameworks. Drug Saf. 2015;38(3):219–32. doi: 10.1007/s40264-015-0278-8 25749722 PMC4374117

[21] Tieu C, Breder CD. A critical evaluation of safety signal analysis using algorithmic standardised MedDRA queries. Drug Saf. 2018;41(12):1375–85. doi: 10.1007/s40264-018-0706-7 30112728

[22] Salim H, Jones AM. Angiotensin II receptor blockers (ARBs) and manufacturing contamination: a retrospective National Register Study into suspected associated adverse drug reactions. Br J Clin Pharmacol. 2022;88(11):4812–27. doi: 10.1111/bcp.15411 35585835 PMC9796460

[23] Cutroneo PM, Sartori D, Tuccori M, Crisafulli S, Battini V, Carnovale C, et al. Conducting and interpreting disproportionality analyses derived from spontaneous reporting systems. Front Drug Saf Regul. 2024;3:1323057. doi: 10.3389/fdsfr.2023.1323057 40980108 PMC12443087

[24] Slattery J, Alvarez Y, Hidalgo A. Choosing thresholds for statistical signal detection with the proportional reporting ratio. Drug Saf. 2013;36(8):687–92. doi: 10.1007/s40264-013-0075-1 23754759

[25] Rothman KJ, Lanes S, Sacks ST. The reporting odds ratio and its advantages over the proportional reporting ratio. Pharmacoepidemiol Drug Saf. 2004;13(8):519–23. doi: 10.1002/pds.1001 15317031

[26] SIDER side effect resource. Accessed 2025 April 10. http://sideeffects.embl.de/

[27] Denisko D, Hoffman MM. Classification and interaction in random forests. Proc Natl Acad Sci U S A. 2018;115(8):1690–2. doi: 10.1073/pnas.1800256115 29440440 PMC5828645

[28] Hoang T, Liu J, Roughead E, Pratt N, Li J. Supervised signal detection for adverse drug reactions in medication dispensing data. Comput Methods Programs Biomed. 2018;161:25–38. doi: 10.1016/j.cmpb.2018.03.021 29852965

[29] Chen X, Shi H, Yang F, Yang L, Lv Y, Wang S, et al. Large-scale identification of adverse drug reaction-related proteins through a random walk model. Sci Rep. 2016;6:36325. doi: 10.1038/srep36325 27805066 PMC5090865

[30] Speiser JL. A random forest method with feature selection for developing medical prediction models with clustered and longitudinal data. J Biomed Inform. 2021;117:103763. doi: 10.1016/j.jbi.2021.103763 33781921 PMC8131242

[31] Li J, Tian Y, Zhu Y, Zhou T, Li J, Ding K, et al. A multicenter random forest model for effective prognosis prediction in collaborative clinical research network. Artif Intell Med. 2020;103:101814. doi: 10.1016/j.artmed.2020.101814 32143809

[32] EsmailyH, TayefiM, DoostiH, Ghayour-MobarhanM, NezamiH, AmirabadizadehA. A comparison between decision tree and random forest in determining the risk factors associated with type 2 diabetes. J Res Health Sci. 2018;18(2):e00412. 29784893

[33] Couronné R, Probst P, Boulesteix A-L. Random forest versus logistic regression: a large-scale benchmark experiment. BMC Bioinform. 2018;19(1):270. doi: 10.1186/s12859-018-2264-5 30016950 PMC6050737

[34] Nwanosike EM, Conway BR, Merchant HA, Hasan SS. Potential applications and performance of machine learning techniques and algorithms in clinical practice: asystematic review. Int J Med Inform. 2022;159:104679. doi: 10.1016/j.ijmedinf.2021.104679 34990939

[35] Random forest classifier. Accessed 2025 April 8. https://scikit-learn/stable/modules/generated/sklearn.ensemble.RandomForestClassifier.html

[36] Khalilia M, Chakraborty S, Popescu M. Predicting disease risks from highly imbalanced data using random forest. BMC Med Inform Decis Mak. 2011;11:51. doi: 10.1186/1472-6947-11-51 21801360 PMC3163175

[37] Santiso S, Casillas A, Pérez A. The class imbalance problem detecting adverse drug reactions in electronic health records. Health Informatics J. 2019;25(4):1768–78. doi: 10.1177/1460458218799470 30230408

[38] Denck J, Ozkirimli E, Wang K. Machine-learning-based adverse drug event prediction from observational health data: a review. Drug Discov Today. 2023;28(9):103715. doi: 10.1016/j.drudis.2023.103715 37467879

[39] Chawla NV, Bowyer KW, Hall LO. SMOTE: synthetic minority over-sampling technique. J Artif Int Res. 2002;16:321–57.

[40] Batista GEAPA, Prati RC, Monard MC. A study of the behavior of several methods for balancing machine learning training data. SIGKDD Explor Newsl. 2004;6(1):20–9. doi: 10.1145/1007730.1007735

[41] Shahriari B, Swersky K, Wang Z, Adams RP, de Freitas N. Taking the Human Out of the Loop: A Review of Bayesian Optimization. Proc IEEE. 2016;104(1):148–75. doi: 10.1109/jproc.2015.2494218

[42] Breiman L. Random forests. Mach Learn. 2001;45:5–32.

[43] Piñero J, Bravo À, Queralt-Rosinach N, Gutiérrez-Sacristán A, Deu-Pons J, Centeno E, et al. DisGeNET: a comprehensive platform integrating information on human disease-associated genes and variants. Nucleic Acids Res. 2017;45(D1):D833–9. doi: 10.1093/nar/gkw943 27924018 PMC5210640

[44] Seatgeek. The fuzz. Accessed 2025 May 1. https://github.com/seatgeek/thefuzz

[45] Anatomical Therapeutic Chemical (ATC) Classification. Accessed 2025 August 4. https://www.who.int/tools/atc-ddd-toolkit/atc-classification

[46] Aronson JK. Medication errors: definitions and classification. Br J Clin Pharmacol. 2009;67(6):599–604. doi: 10.1111/j.1365-2125.2009.03415.x 19594526 PMC2723196

[47] Patadia VK, Coloma P, Schuemie MJ, Herings R, Gini R, Mazzaglia G, et al. Using real-world healthcare data for pharmacovigilance signal detection - the experience of the EU-ADR project. Expert Rev Clin Pharmacol. 2015;8(1):95–102. doi: 10.1586/17512433.2015.992878 25487079

[48] Blonde L, Khunti K, Harris SB, Meizinger C, Skolnik NS. Interpretation and impact of real-world clinical data for the practicing clinician. Adv Ther. 2018;35(11):1763–74. doi: 10.1007/s12325-018-0805-y 30357570 PMC6223979

[49] Hazell L, Shakir SAW. Under-reporting of adverse drug reactions : a systematic review. Drug Saf. 2006;29(5):385–96. doi: 10.2165/00002018-200629050-00003 16689555

[50] García-Abeijon P, Costa C, Taracido M, Herdeiro MT, Torre C, Figueiras A. Factors associated with underreporting of adverse drug reactions by health care professionals: a systematic review update. Drug Saf. 2023;46(7):625–36. doi: 10.1007/s40264-023-01302-7 37277678 PMC10279571

[51] Rolfes L, van Hunsel F, Wilkes S, van Grootheest K, van Puijenbroek E. Adverse drug reaction reports of patients and healthcare professionals-differences in reported information. Pharmacoepidemiol Drug Saf. 2015;24(2):152–8. doi: 10.1002/pds.3687 25079444

[52] van Hunsel F, Passier A, van Grootheest K. Comparing patients’ and healthcare professionals’ ADR reports after media attention: the broadcast of a Dutch television programme about the benefits and risks of statins as an example. Br J Clin Pharmacol. 2009;67(5):558–64. doi: 10.1111/j.1365-2125.2009.03400.x 19552751 PMC2686073

[53] Blehar MC, Spong C, Grady C, Goldkind SF, Sahin L, Clayton JA. Enrolling pregnant women: issues in clinical research. Womens Health Issues. 2013;23(1):e39-45. doi: 10.1016/j.whi.2012.10.003 23312713 PMC3547525

[54] Chen MS Jr, Lara PN, Dang JHT, Paterniti DA, Kelly K. Twenty years post-NIH Revitalization Act: enhancing minority participation in clinical trials (EMPaCT): laying the groundwork for improving minority clinical trial accrual: renewing the case for enhancing minority participation in cancer clinical trials. Cancer. 2014;120 Suppl 7(0 7):1091–6. doi: 10.1002/cncr.28575 24643646 PMC3980490

[55] Pardhan S, Sehmbi T, Wijewickrama R, Onumajuru H, Piyasena MP. Barriers and facilitators for engaging underrepresented ethnic minority populations in healthcare research: an umbrella review. Int J Equity Health. 2025;24(1):70. doi: 10.1186/s12939-025-02431-4 40075407 PMC11905581

[56] Dey T, Widmer M, Coomarasamy A, Goudar SS, Berrueta M, Coutinho E, et al. Advancing maternal and perinatal health through clinical trials: key insights from a WHO global consultation. Lancet Glob Health. 2025;13(4):e740–8. doi: 10.1016/S2214-109X(24)00512-6 40155111

[57] Corrigan OP. A risky business: the detection of adverse drug reactions in clinical trials and post-marketing exercises. Soc Sci Med. 2002;55(3):497–507. doi: 10.1016/s0277-9536(01)00183-6 12144155

[58] Diao G, Liu GF, Zeng D, Wang W, Tan X, Heyse JF, et al. Efficient methods for signal detection from correlated adverse events in clinical trials. Biometrics. 2019;75(3):1000–8. doi: 10.1111/biom.13031 30690717 PMC6661211

[59] Ioannidis JPA. Why most clinical research is not useful. PLoS Med. 2016;13:e1002049. 10.1371/journal.pmed.1002049 PMC4915619 27328301

[60] Halevy A, Norvig P, Pereira F. The unreasonable effectiveness of data. IEEE Intell Syst. 2009;24(2):8–12. doi: 10.1109/mis.2009.36

[61] Simon JB, Karkada D, Ghosh N. More is better in modern machine learning: when infinite overparameterization is optimal and overfitting is obligatory. Epub ahead of print. 2024. doi: 10.48550/arXiv.2311.14646

[62] Wu J, Chen XY, Zhang H. Hyperparameter optimization for machine learning models based on bayesian optimization. J Electron Sci Technol. 2019;17:26–40.

[63] Miles MF, Barhite S, Sganga M, Elliott M. Phosducin-like protein: an ethanol-responsive potential modulator of guanine nucleotide-binding protein function. Proc Natl Acad Sci U S A. 1993;90(22):10831–5. doi: 10.1073/pnas.90.22.10831 8248177 PMC47872

[64] Aagaard L, Hansen EH. Adverse drug reactions reported by consumers for nervous system medications in Europe 2007 to 2011. BMC Pharmacol Toxicol. 2013;14:30. doi: 10.1186/2050-6511-14-30 23763896 PMC3685574

[65] Wu L, Ingle T, Liu Z, Zhao-Wong A, Harris S, Thakkar S, et al. Study of serious adverse drug reactions using FDA-approved drug labeling and MedDRA. BMC Bioinform. 2019;20(Suppl 2):97. doi: 10.1186/s12859-019-2628-5 30871458 PMC6419320

[66] Aagaard L, Hansen EH. Adverse drug reactions from psychotropic medicines in the paediatric population: analysis of reports to the Danish Medicines Agency over a decade. BMC Res Notes. 2010;3:176. doi: 10.1186/1756-0500-3-176 20573185 PMC2901212

[67] Gao Y, Zhang X, Sun Z, Chandak P, Bu J, Wang H. Precision adverse drug reactions prediction with heterogeneous graph neural network. Adv Sci (Weinh). 2024;12(4):e2404671. doi: 10.1002/advs.202404671 39630592 PMC11775569

[68] Kaur H, Pannu HS, Malhi AK. A systematic review on imbalanced data challenges in machine learning. ACM Comput Surv. 2019;52(4):1–36. doi: 10.1145/3343440

[69] Goyal M, Mahmoud QH. A systematic review of synthetic data generation techniques using generative AI. Electronics. 2024;13(17):3509. doi: 10.3390/electronics13173509

