# Supplementary material for: An interpretable machine learning framework for adverse drug reaction prediction from drug-target interactions
Source: PLoS One. 2026 Jan 30;21(1):e0340900. doi: 10.1371/journal.pone.0340900 (PMC12858017; doi:10.1371/journal.pone.0340900)
Supplement: S1 Appendix — Tables (Table 1 – Model Prediction Values, Table 2 – Random Forest Metrics Results, Table 3 – Decision Tree Model Metrics, Table 4 -t-test values for Decision Tree and Random Forest comparison of model metrics, and Table 5 – Drugs of Interest ATC codes. (PDF) [file pone.0340900.s001.pdf]

SUPPORTING INFORMATION

An Interpretable Machine Learning Framework for Adverse Drug Reaction Prediction from Drug-Target Interactions

Joseph Roberts-Nuttall,[1*] Alan M. Jones,[2*] Marco Castellani,[1] and Duc Pham[1]

[1] School of Mechanical Engineering, University of Birmingham, Edgbaston, B15 2TT, United Kingdom

[2] School of Pharmacy, University of Birmingham, Edgbaston, B15 2TT, United Kingdom

*Corresponding authors: JRN, AMJ

S1 Appendix

Additional Methods

Disproportionality Analysis

Equations:

	Adverse Event of Interest (Y)	Other Adverse Events	Total
Using Drug of Interest (X)	A	B	(A+B)
Using Other Drugs	C	D	(C+D)
Total	(A+C)	(B+D)	(A+B+C+D)

$$\boldsymbol{ROR} = \frac{\left(A/_C\right)}{\left(B/_D\right)}$$

$$\boldsymbol{PRR} = \frac{\left(A/_{(A+B)}\right)}{\left(C/_{(C+D)}\right)}$$

CI Lower:

In addition to the ROR score, a statistical significance was calculated. This threshold was used for the final data and not in the comparison, since the threshold was not appropriate when using percentages.

$$CI_{lower} = e^{ln(ROR) - 1.96 \cdot SE_{ln(ROR)}}$$

Where ROR is the Reporting Odds Ratio, $SE_{ln(ROR)}$ is the standard error of the natural logarithm for ROR, and 1.96 corresponds to the z-score for a 95% confidence level under a normal distribution.

Jaccard Index

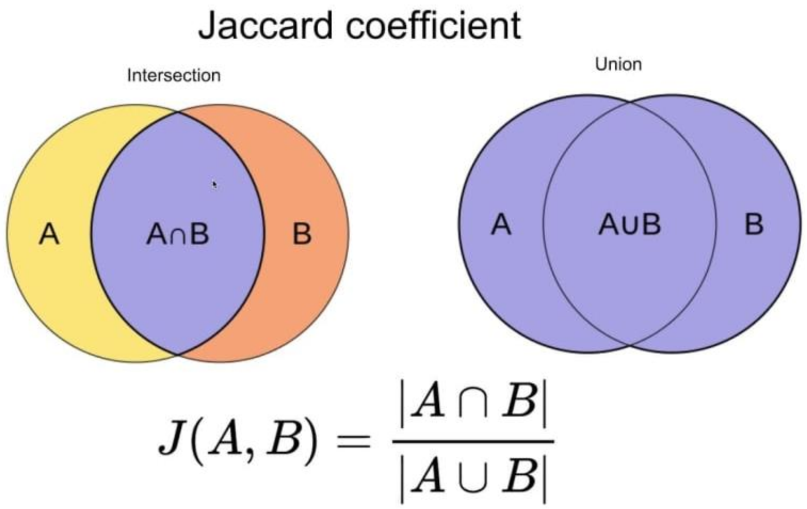

Available from: [Benchmarking of Textual Models - Jaccard Similarity - DEV Community]

Performance Metrics

Where TP is True positive, TN is True Negative, FP is False Positive, FN is False Negative, TPR is True Positive Rate and FPR is False Positive Rate

Accuracy

$$\frac{TP + TN}{TP + TN + FP + FN}$$

ROC AUC

$$\int_{x=0}^{1} TPR\left(FPR^{-1}(x)\right)dx$$

Precision

$$\frac{TP}{TP + FP}$$

Recall

$$\frac{TP}{TP + FN}$$

F1-Score

$$\frac{2 \times precision \times recall}{precision + recall}$$

MCC

$$\frac{TP \times TN - FP \times FN}{\sqrt{(TP + FP)(TP + FN)(TN + FP)(TN + FN)}}$$

TABLES

Table 1. Model Prediction Values

SOC Category	TP	TN	FP	FN	TP%	TN%	FP%	FN%
**Blood**	8	217	7	18	3.2	86.8	2.8	7.2
**Card**	10	212	12	16	4.0	84.8	4.8	6.4
**Cong**	4	209	22	15	1.6	83.6	8.8	6.0
**Ear**	3	213	14	20	1.2	85.2	5.6	8.0
**Endo**	7	221	12	10	2.8	88.4	4.8	4.0
**Eye**	5	209	22	14	2.0	83.6	8.8	5.6
**Gastr**	0	226	4	20	0.0	90.4	1.6	8.0
**Hepat**	14	188	32	16	5.6	75.2	12.8	6.4
**Immun**	0	220	1	29	0.0	88.0	0.4	11.6
**Infec**	4	220	12	14	1.6	88.0	4.8	5.6
**Metab**	7	219	10	14	2.8	87.6	4.0	5.6
**Musc**	1	235	6	8	0.4	94.0	2.4	3.2
**Neopl**	9	219	3	19	3.6	87.6	1.2	7.6
**Nerv**	5	224	12	9	2.0	89.6	4.8	3.6
**Preg**	3	231	3	13	1.2	92.4	1.2	5.2

**Psych**	6	228	13	3	2.4	91.2	5.2	1.2
**Renal**	2	216	10	22	0.8	86.4	4.0	8.8
**Repro**	10	218	11	11	4.0	87.2	4.4	4.4
**Resp**	4	218	8	20	1.6	87.2	3.2	8.0
**Skin**	1	223	3	23	0.4	89.2	1.2	9.2
**Vasc**	4	213	11	22	1.6	85.2	4.4	8.8

Table 2. Random Forest Metrics Results

SOC Category	ROC AUC	Accuracy	Precision	Recall	F1 Score	MCC
**Psych**	0.94	0.94	0.32	0.67	0.43	0.43
**Nerv**	0.90	0.92	0.29	0.36	0.32	0.28
**Neopl**	0.87	0.91	0.75	0.32	0.45	0.45
**Preg**	0.85	0.94	0.50	0.19	0.27	0.28
**Endo**	0.84	0.91	0.37	0.41	0.39	0.34
**Blood**	0.81	0.90	0.53	0.31	0.39	0.36
**Repro**	0.81	0.91	0.48	0.48	0.48	0.43
**Metab**	0.81	0.90	0.41	0.33	0.37	0.32
**Infec**	0.80	0.90	0.25	0.22	0.24	0.18
**Gastr**	0.78	0.90	0.00	0.00	0.00	-0.04
**Card**	0.76	0.89	0.45	0.38	0.42	0.36
**Musc**	0.76	0.94	0.14	0.11	0.13	0.10
**Eye**	0.73	0.86	0.19	0.26	0.22	0.14
**Resp**	0.72	0.89	0.33	0.17	0.22	0.18
**Skin**	0.72	0.90	0.25	0.04	0.07	0.07
**Vasc**	0.71	0.87	0.27	0.15	0.20	0.13
**Hepat**	0.69	0.81	0.30	0.47	0.37	0.27
**Renal**	0.68	0.87	0.17	0.08	0.11	0.05
**Cong**	0.66	0.85	0.15	0.21	0.18	0.10
**Immun**	0.65	0.88	0.00	0.00	0.00	-0.02
**Ear**	0.64	0.86	0.18	0.13	0.15	0.08

Table 3. Decision Tree Model Metrics

SOC Category	ROC AUC	Accuracy	Precision	Recall	F1 Score	MCC
Psych	0.85	0.86	0.14	0.56	0.22	0.23
Preg	0.81	0.92	0.39	0.44	0.41	0.37
Metab	0.77	0.88	0.30	0.29	0.29	0.23
Neopl	0.77	0.83	0.34	0.54	0.42	0.34
Endo	0.77	0.90	0.36	0.53	0.43	0.39
Repro	0.77	0.89	0.38	0.48	0.43	0.37
Nerv	0.71	0.90	0.25	0.36	0.29	0.25
Card	0.70	0.82	0.26	0.38	0.31	0.21
Blood	0.70	0.85	0.20	0.15	0.17	0.09
Vasc	0.68	0.86	0.30	0.23	0.26	0.19
Gastr	0.68	0.80	0.14	0.30	0.19	0.10
Infec	0.67	0.89	0.19	0.17	0.18	0.12

Resp	0.65	0.85	0.22	0.21	0.21	0.13
Skin	0.65	0.54	0.14	0.75	0.24	0.16
Cong	0.63	0.87	0.25	0.37	0.30	0.23
Renal	0.62	0.84	0.25	0.33	0.29	0.20
Eye	0.62	0.84	0.13	0.21	0.16	0.08
Prod	0.62	0.86	0.19	0.26	0.22	0.15
Musc	0.60	0.86	0.07	0.22	0.11	0.06
Ear	0.60	0.85	0.17	0.17	0.17	0.09
Hepat	0.60	0.88	0.57	0.13	0.22	0.24
Immun	0.59	0.83	0.00	0.00	0.00	-0.09

Table 4. t-test values for Decision Tree and Random Forest comparison of model metrics

Metric	t-statistic	p-value	Significant at 0.05?
ROC AUC	-3.257	0.002	TRUE
Accuracy	-2.538	0.017	TRUE
Precision	-1.268	0.213	FALSE
Recall	1.338	0.189	FALSE
F1 Score	-0.113	0.911	FALSE
MCC	-0.580	0.565	FALSE

Table 5. Drugs of interest ATC Codes

Drug	ATC Code
**Pizotifen**	N02CX01
**Asenapine**	N05AH05
**Rotigotine**	N04BC09
**Diazepam**	N05BA01
**Galantamine**	N06DA04
**Clonazepam**	N03AE01
**Nitrazepam**	N05CD02
**Oxazepam**	N05BA04
**Paliperidone**	N05AX13
**Temazepam**	N05CD07
**Levodopa**	N04BA01
**Pergolide**	N04BC02
**Ketazolam**	N05BA10
**Venlafaxine**	N06AX16
**Cariprazine**	N05AX15
**Paroxetine**	N06AB05
**Ketamine**	N01AX03
**Pentazocine**	N02AD01
**Agomelatine**	N06AX22
**Lurasidone**	N05AE05
**Flurazepam**	N05CD01

**Lysuride**	G02CB02, N02CA07
**Citalopram**	N06AB04
**Zolpidem**	N05CF02
**Moclobemide**	N06AG02
**Ethanol**	D08AX08, V03AB16, V03AZ01
**Donepezil**	N06DA02
**Zotepine**	N05AX11
**Melatonin**	N05CH01
**Nicotine**	N07BA01
**Cabergoline**	G02CB03, N04BC06
**Ketamine S-Isomer**	N06AX27, N01AX14
**Cyproheptadine**	R06AX02
**Promethazine**	R06AD52, D04AA10, R06AD02
**Sertraline**	N06AB06
**Bromazepam**	N05BA08
**Mirtazapine**	N06AX11
**Escitalopram**	N06AB10
**Benztropine**	N04AC01
**Buspirone**	N05BE01
**Vortioxetine**	N06AX26
**Fluoxetine**	N06AB03
**Flunitrazepam**	N05CD03
**Ropinirole**	N04BC04
**Aripiprazole**	N05AX12
**Triazolam**	N05CD05
**Pramipexole**	N04BC05
**Dexamphetamine**	N06BA02
**Methylphenidate**	N06BA04
